# Evaluating the value of a web-based natural medicine clinical decision tool at an academic medical center

**DOI:** 10.1186/1472-6963-11-279

**Published:** 2011-10-19

**Authors:** Sue Boehmer, Kelly Karpa

**Affiliations:** 1 Department of Public Health Sciences, Division of Biostatistics, Penn State University College of Medicine, 500 University Drive, Hershey, PA 17033, USA; 2 Department of Pharmacology, Penn State University College of Medicine, 500 University Drive Hershey, PA 17033, USA

## Abstract

**Background:**

Consumer use of herbal and natural products (H/NP) is increasing, yet physicians are often unprepared to provide guidance due to lack of educational training. This knowledge deficit may place consumers at risk of clinical complications. We wished to evaluate the impact that a natural medicine clinical decision tool has on faculty attitudes, practice experiences, and needs with respect to H/NP.

**Methods:**

All physicians and clinical staff (nurse practitioners, physicians assistants) (n = 532) in departments of Pediatrics, Family and Community Medicine, and Internal Medicine at our medical center were invited to complete 2 electronic surveys. The first survey was completed immediately before access to a H/NP clinical-decision tool was obtained; the second survey was completed the following year.

**Results:**

Responses were obtained from 89 of 532 practitioners (16.7%) on the first survey and 87 of 535 (16.3%) clinicians on the second survey. Attitudes towards H/NP varied with gender, age, time in practice, and training. At baseline, before having an evidence-based resource available, nearly half the respondents indicated that they rarely or never ask about H/NP when taking a patient medication history. The majority of these respondents (81%) indicated that they would like to learn more about H/NP, but 72% admitted difficulty finding evidence-based information. After implementing the H/NP tool, 63% of database-user respondents indicated that they now ask patients about H/NP when taking a drug history. Compared to results from the baseline survey, respondents who used the database indicated that the tool significantly increased their ability to find reliable H/NP information (P < 0.0001), boosted their knowledge of H/NP (p < 0.0001), and increased their confidence in providing accurate H/NP answers to patients and colleagues (P < 0.0001).

**Conclusions:**

Our results demonstrate healthcare provider knowledge and confidence with H/NP can be improved without costly and time-consuming formal H/NP curricula. Yet, it will be challenging to make providers aware of such resources.

## Background

Use of natural and herbal products (H/NP) has experienced a resurgence in the United States since passing of the Dietary Supplement Health Education Act (DSHEA) in 1994 [1]. Less than two decades ago, only 4,000 H/NP were available in the United States; today, there are more than 30,000 products, with more than 1,000 new options added each year [1,2]. According to DSHEA, dietary supplements include vitamins, minerals, herbs or other botanicals, amino acids, other dietary substances for use by man that are intended to supplement the diet, as well as concentrates, metabolites, constituents, extracts, or combinations of these ingredients. Presently, almost one fifth of the adult population of the United States admits to using non-vitamin, non-mineral dietary supplements [3]. It is estimated that US consumers spend more than $15 billion each year on these products [4].

Given the popularity of H/NP, minimal premarket safety or efficacy requirements, and often unsubstantiated health claims, healthcare providers need to be knowledgeable about many different supplements or have access to evidence-based information that can assist in assessing the risks and benefits of these products. Studies have shown that consumers are often misinformed about the H/NP therapies they are using, and they may be at risk of harm [5-7]. Few users of H/NP tell their physicians about the dietary supplements they are using partly because doctors do not consistently inquire about alternative therapies, and in part, because patients do not perceive healthcare professionals to be knowledgeable about H/NP [8,9]. Additionally, patients do not tell their physicians about the H/NP they use due to fear that their physicians will disapprove [8,9]. Failure to disclose use of dietary supplements may be an important public health issue, yet even when consumers look to healthcare providers for guidance with dietary supplements, physicians are frequently unprepared to effectively handle the clinical questions that arise. Lack of formal training and inadequate or conflicting information from manufacturers contributes to physician reluctance to engage in discussions of H/NP, and physicians' knowledge in the area of H/NP is lacking. It has previously been shown that physicians dramatically under-estimate the potential for these therapies to cause side effects and interact with conventional medications [10]. Understandably physicians may be hesitant to discuss H/NP when they themselves are uncertain about the safety and efficacy of these products [2]. As a result, many physicians disavow use of all H/NP or take a "don't ask, don't tell" approach toward the supplements [11,12]. Yet, this tactic only alienates patients who will likely use H/NP anyway, but will subsequently avoid such discussions with their healthcare provider. It has previously been shown that well-informed physicians are more confident in patient interactions, and that alone improves healthcare quality [13,14]. Thus, it is imperative that physicians receive some education on the H/NP related to their practice or at least have access to evidence-based information when patient questions arise.

Unlike well-established alternative medicine centers featured at some hospitals [15-17], our medical center had no complementary and alternative resources established at the time this study was conceived. Therefore, our initial objective was to assess attitudes, knowledge, professional behaviors, and needs of clinicians with respect to H/NP. Once we recognized the needs of our providers in this area, our second objective was to determine the extent to which an H/NP clinical decision tool could meet those needs by providing evidence-based H/NP information accessible at the point of care. We hypothesized that a web-based tool would meet the needs that our clinicians had expressed with regard to H/NP.

## Methods

### Surveys

Two surveys were prepared for physicians and clinical staff (nurse practitioner, physician assistants) practicing within the departments of Pediatrics (Peds), Family and Community Medicine (FCM), and Internal Medicine (Med) at the Penn State Milton S. Hershey Medical Center and College of Medicine. This is an academic medical center located in central Pennsylvania. Comprised of 484 licensed beds, the institution is also home to a cancer institute with a catchment area of 27 counties, many of them underserved, as well as the region's only level 1 pediatric trauma center. A founding focus of the medical school was primary care, a situation that remains evident even today by 35% of students entering primary care residencies after graduation.

E-mails inviting clinicians to complete the surveys were sent from the academic offices of departmental chairman of Peds and FCM and from the offices of all 9 division chiefs within the department of Med. These emails asked all physicians (faculty and residents) and clinical staff (nurse practitioners and physician assistants) to complete surveys about knowledge, attitudes, and experiences with H/NP. The emails contained a direct link to the surveys, which were developed using SurveyMonkey such that the data could be collected and stored anonymously. Responses from nurse practitioners and physician assistants were included in our dataset since these allied healthcare providers play key roles in patient care, thus the terms "provider" and "clinician" in our data analysis refer to any of the physicians or allied health care providers that are licensed as MDs, DOs, PAs, and NPs.

The nature of each survey was explained in the emails and again when physicians accessed the link. Willingness to contribute to this endeavor was indicated by providers' response to the first question of each survey which asked for a "yes" response if they agreed to participate. Any surveys in which a "no" response was chosen to this initial question were considered "unusable" since consent had not been given for participation. After the initial email was sent inviting clinician participation, three additional reminders were mailed every 10 days over the following month to encourage survey completion. The *de novo *surveys were initially piloted by three residents and two FCM physicians that lead our campus' Practice Based Research Network (PBRN); approval of the PBRN was necessary prior to distributing the survey. Several questions were modified to enhance clarity of responses on the basis of this trial.

The first survey was comprised of a total of 48 questions that were estimated to require 15 minutes to complete. At the beginning of the survey, H/NP were defined as: supplements containing plant-based herbals (e.g., Echinacea, St. John's Wort, saw palmetto, garlic, ginger, gingko, etc.), amino acids and their derivatives, probiotics, fish oils, and enzymes. On the other hand, clinicians were specifically directed *not *to include multivitamin products and minerals such as calcium, iron, or folate in the H/NP definition. The first survey began with 11 demographic questions, followed by 26 questions assessing attitudes and practice experiences with respect to H/NP, and the survey concluded with ten multiple choice questions assessing knowledge about safety and efficacy of commonly used H/NPs. The first survey was conducted in November and December, 2008.

The following year, the same clinical providers (MD, DO, NP, PA) from the 3 departments were invited to participate in a second survey, which was comprised of ten demographic questions and twelve questions that aimed to assess the utility of the NMCD, as well as changes in opinions or practice experiences with respect to H/NP that had occurred as a result of having access to the electronic resource. Residents received this second survey in May and June to capture their responses prior to residency completion; the other providers received the second survey in November and December of 2009. It was estimated that 5-10 minutes was adequate for completing the second survey. No incentives were offered for completing either survey. The study was granted exemption from oversight by the Investigational Review Board of the Penn State University Milton S. Hershey Medical Center (#29014EM; 8-4-2008) since data was collected in an anonymous fashion.

### Database

Immediately following the first survey, a subscription to a web-based clinical decision H/NP tool (Natural Medicines Comprehensive Database (NMCD), Stockton, CA) was procured for the 2009 calendar year. The number of "hits" that originated from Penn State - Hershey internet protocol (IP) addresses was recorded by NMCD.

In the first six months after subscribing to the NMCD, a number of demonstrations were delivered across institutional divisions to heighten awareness of the resource. Specifically, oral demonstrations were delivered at a Faculty Organization meeting, as well as faculty meetings for the Med divisions, a FCM faculty meeting, and a basic science departmental faculty meeting. Additional demonstrations of the electronic tool took place at staff meetings for both nurse practice counsel and nurse managers. The NMCD was also "advertised" in the campus newsletter, and featured as the main topic of the campus intranet for several weeks. In addition, the NMCD was prominently displayed and demonstrated at several physician and nursing technology fairs on campus and added to the FCM toolbar within the patient electronic medical record. Furthermore, special faculty seminars were jointly conducted between staff librarians and pharmacology faculty to demonstrate the clinical utility of the database to other allied health professionals, including dieticians. Finally, three *de novo *electronic tutorials were created and prominently posted on the campus library's website to illustrate different features of the database and the applicability of the tool to clinical practice. http://www.pennstatehershey.org/web/library/resources/natural-medicines-comprehensive-database. Additionally, during the inaugural year in which the NMCD was made available on our campus, numerous emails targeting Med, FCM, and Ped providers were sent reminding of the electronic tool.

### Statistical Analysis

Initially, we hypothesized that clinicians would have unmet educational needs with regard to H/NP in patient encounters. Subsequently, we further surmised that an electronic clinical decision support tool would help to meet these needs. The statistical software package, SAS version 9.1 (copyright by SAS Institute, Cary, NC), was used to analyze the data. Descriptive statistics were generated including means, medians, and standard deviations for continuous variables and frequency tables for categorical variables. Differences between groups were characterized using contingency table analysis; significance levels were determined by Pearson's chi-square statistics and Fisher's Exact tests. Significance was set at p < 0.05.

## Results

### Baseline Attitudes, Practice Experiences, and Knowledge with Herbal and Natural Products

**Figure 1:**
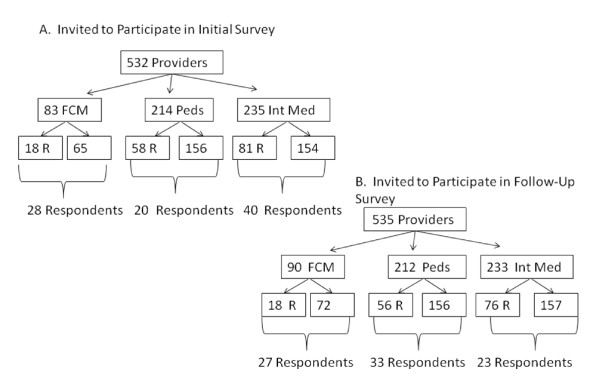
**Providers Invited to Participate in Herbal and Natural Product Assessment**. (A) Initial Baseline Survey to assess H/NP attitudes, experiences and knowledge in 2008. (B) Second Follow-up Survey to assess the extent the electronic tool changed attitudes, experiences, and knowledge, 2009. FCM, Department of Family and Community Medicine; Peds, Department of Pediatrics; Int Med, Department of Internal Medicine; R, residents.

A total of 532 providers, including 157 residents, were invited to participate in the initial survey assessing needs, attitudes, experiences, and knowledge of H/NP (Figure 1). Responses were obtained from 89 of these practitioners (16.7%). In addition to attending physicians, fourteen resident physicians responded, as well as ten nurse practitioners and five physician's assistants (collectively referred to as respondents, providers, or clinicians herein). The cohort of respondents was comprised of 20 individuals from the pediatric division, 40 providers from internal medicine, and 28 providers from FCM. Based upon the sizes of these clinical departments, the highest response rate was obtained from FCM, with 34% replying to the survey. In contrast, lower responses rates of 10% and 24% came from the departments of pediatrics and internal medicine, respectively. Demographic characteristics of the respondents are depicted in Table 1.

**Table 1:** Demographics of Survey Respondents

Responders to the Baseline Needs Assessment Survey in 2008	N (%)	Responders to Follow-up Survey in 2009	N (%)	p-value
Gender		Gender		0.60
Male	46 (52)	Male	41 (48)	
Female	43 (48)	Female	45 (52)	
Age (years)		Age		0.68
≤ 30	14 (16)	≤ 30	16 (18)	
31-40	23 (26)	31-40	22 (25)	
41-50	20 (22)	41-50	22 (25)	
51-60	26 (30)	51-60	18 (21)	
≥ 60	6 (7)	≥ 60	9(10)	
Ethnicity		Ethnicity		0.49
Asian	11 (13)	Asian	10(12)	
Bi-racial	1 (1)	Bi-racial	0	
Black	0	Black	2(2)	
Caucasian	72 (83)	Caucasian	69(81)	
Hispanic	0	Hispanic	2(2)	
Other	1 (1)	Do not wish to answer	2(2)	
Place of Training		Place of Training		0.75
Inside USA	76 (85)	Inside USA	74(87)	
Outside USA	13 (15)	Outside USA	11(13)	
Provider's Medical Degree		Medical Degree		0.12
MD	68 (76)	MD	69 (79)	
DO	6 (7)	DO	5 (6)	
NP	10 (11)	NP	5 (6)	
PA	5 (6)	PA	2 (2)	
		RN	3(4)	
		Others	3 (4)	
Years Since Completion of Training		Years Since Completion of Training		0.66
Still in residency/fellowship	14 (16)	Still in residency/fellowship	18(21)	
Under 5	18(20)	Under 5	11(13)	
6-15	24 (27)	6-15	26(3)	
16-25	21 (24)	16-25	19(22)	
Over 26	12 (13)	Over 26	13(15)	

Demographic characteristics of providers who responded to initial baseline survey and the second follow-up survey are shown. Percentages may be > or < 100% due to rounding or absent responses. MD, medical doctor; DO, doctor of osteopathy; NP, nurse practitioner; PA, physician assistant; others, self-identified as PharmDs, certified diabetic educators, and PhDs

Clinicians readily acknowledged that their patients utilize H/NP, with the majority of respondents estimating that 26 to 50% of their patients are using dietary supplements. Despite recognizing that H/NP use is common among their patients, however, nearly half of the respondents (41%) indicated that they "never" or "rarely" ask about H/NP when taking a patient history. The majority of respondents (71%) estimated that they had discussed H/NP with fewer than 25% of their patients. Moreover, when the topic of H/NP was discussed, the provider initiated the conversation only 37% of the time. Seventy-nine percent of respondents indicated that they "rarely" or "never" recommend H/NP to patients, with the internists least likely of the three clinical divisions to recommend dietary supplements to their patients. Ninety-five percent of internal medicine providers reported that they had "never" or "rarely" recommended H/NP to their patients.

A large majority [n = 80 (92%)] of providers indicated that, as health care practitioners, they should have knowledge or, at least, access to accurate information about common H/NP used by consumers. This attitude was expressed by 100% of both Ped and FCM respondents but only 82% of internists (p = 0.01)(Table 2). Fifty-eight respondents (67%) expressed the opinion that knowledge of H/NP can lead to more successful patient outcomes, but only 43 (49%) clinicians felt that it was their responsibility to counsel and provide information to patients about the H/NP that they are using. Internists were less than half as likely to agree that they should counsel patients about H/NP compared to their Ped and Med colleagues (p = 0.01; Table 2; 12 of 39 [31%] versus 13 of 20 [65%] versus 18 of 28 [64%], respectively).

**Table 2:** Attitudes and Practice Experiences With Herbal and Natural Products

	Gender		Specialty			Degree		Training		Years in Practice			Rank	
	M (%)	F (%)	Ped (%)	Int Med (%)	FCM (%)	MD, DO (%)	NP, PA (%)	In USA (%)	Out of USA (%)	In residency/fellowship or < 5 years post-training (%)	5-15 years (%)	> 15 years (%)	Resident (%)	Board Certified/Eligible (%)
Practitioner knowledge of H/NP leads to better patient outcomes	55	79†	70	59	75	64	80	67	67	75	67	58	72	62
Practitioners should have knowledge or accessibility to information about common H/NP	86	98	100	82*	100	92	93	93	83	97	100	81*	100	90
Some herbal products hold promise for treatment of medical conditions	71	79	90	64	79	74	80	79	50	75	96	58 ξ	79	74
Patient counseling about H/NP is the practitioner's responsibility	39	61 φ	65	31 ψ	64	46	67	51	42	44	50	55	50	47
H/NP rarely cause side effects	81	51*	50	74	68	73	33 ξ	63	91	56	58	83 φ	57	75
H/NP rarely interact with prescription drugs	67	47	45	63	56	63	27†	54	73	47	50	72	36	68
Would like to learn more about H/NP	72	91 φ	85	74	89	77	100	83	73	91	88	67††	93	74
Would feel more confident discussing H/NP with formal training	81	91	90	82	89	85	93	88	73	94	92	73††	93	82
Would feel more confident discussing H/NP with access to a database that contained product monographs	93	95	95	95	93	93	100	96	82	94	100	90	85	95
It is easy for me to find reliable information about H/NP	37	19	30	13*	46	29	20	29	18	25	17	40	21	32
Would absolutely use an evidence-based tool to evaluate H/NP if one was available	50	76 **	79	59	57	59	80	62	67	55	75	61	54	60
Always ask patients about H/NP when taking a drug history	11	35*	25	23	21	21	33	23	25	13	38	23	14	21
When my patients and I discuss H/NP, I usually initiate the conversation	36	37	35	41	32	35	47	41	8†	25	42	45	14	40
Often recommend H/NP to friends or relatives	2	16	21	3	11	9	13	9	8	6	9	13	7	9
Have personally used an H/NP within the past month	30	28	40	23	29	28	33	29	25	31	21	32	36	24

Provider attitudes and practice experiences with herbal and natural products vary with gender, specialty, degree, country of training, years in practice, and rank achieved. * p = 0.01; † p = 0.02; **p = 0.03; ††p = 0.04; φ p = 0.05; ξ p = 0.006; ψ p = 0.007

In addition to differences in attitudes among clinicians of different specialties, differences in H/NP attitudes were also observed between providers of different genders, residents versus attending faculty, those who have been practicing the longest versus the shortest, physicians (MD, DO) versus providers with other degrees (NP, PA), and those trained in the USA versus other countries (Table 2). Female practitioners indicated a greater likelihood than their male counterparts to inquire about H/NP when taking a drug history (p = 0.01)(Table 2), and they exhibited a heightened awareness of interactions and side effects (p = 0.01) that H/NP can cause. Residents, on the other hand, were less cognizant compared to attending faculty (p = 0.04) of the potential for H/NP to interact with prescription drugs and cause side effects. Residents were also less likely to ask patients about H/NP use. Non-physician providers were less aware of the potential for H/NP to cause side effects (p = 0.006) and drug interactions (p = 0.02), but also tended to be more interested in acquiring additional education about H/NP compared with physicians (Table 2). Completion of medical training outside the USA was also associated with a tendency toward having a favorable view of the benefits of H/NP as compared to their potential to cause side effects (Table 2).

Overall, the majority of clinicians (n = 70; 81%) stated that they would like to learn more about H/NP. The majority (63%) admitted that they know relatively little about this topic, and only 10% expressed confidence in their ability to accurately answer patient's questions about H/NP.

To explore familiarity with common H/NP in ordinary clinical scenarios, ten knowledge-based multiple-choice questions modified from Shapiro [2] were included within the survey. In addition to a correct answer and a series of incorrect answers, an "I don't know" option was also included. This option was added in attempt to keep providers from guessing at answers they did not know with certainty. Overall, providers in FCM achieved a greater proportion of correct responses compared to the other two clinical departments for most (eight) knowledge questions (data not shown). Recognizing their knowledge deficit, 74 respondents (86%) admitted that they would feel more confident discussing H/NP with patients if they had more education in this area. Furthermore, most (n = 80) practitioners (94%) indicated that they would feel more confident discussing H/NP with patients if they had access to an evidence-based tool that contained product monographs.

In this baseline assessment, the majority of survey respondents (n = 62; 72%) admitted that they find it difficult to find reliable information about H/NP. Many (n = 54; 63%) responded that they would "absolutely" use an evidence-based, electronic tool to evaluate safety or efficacy of H/NP if one was made available through the university. Only one individual indicated that such an instrument would "definitely not" be of interest to them. Fifty-seven respondents (66%) indicated that their motivation for using such a tool would be to recommend H/NP that are safe and effective, and 45 respondents (52%) indicated that dissuading patients from using unsafe or ineffective H/NP was important to them.

### Follow-Up Assessment: Second Survey

After having the NMCD available for six months, residents in the departments of Ped, Med, and FCM were again asked to participate in a follow-up assessment regarding their views pertaining to the utility of the H/NP tool in their practice. Other clinical providers in these departments were surveyed a year after the first survey had been distributed. In total, 535 providers, including 150 residents were invited to participate in the second survey (Figure 1). Responses were obtained from 87 individuals (16.3%), resulting in a similar response rate to that attained by the initial survey. Respondents who indicated their departmental affiliations represented Ped (n = 33), Med (n = 23), FCM (n = 27), as well as clinical psychology (n = 1) and the pharmacy (n = 1) (responses from these later 2 providers were not included in subsequent data analysis). Overall the demographic characteristics of those that responded to the baseline survey and the follow-up survey were similar (Table 1); however, the two groups of respondents were not identical. Thirty-three individuals (39%) indicated that they had responded to the baseline survey, but 21 (24%) indicated they had not participated previously, and 32 (37%) admitted that they could not remember. When the number of respondents was considered as a function of departmental size, the highest response rate (30%) was again obtained from providers in FCM.

Of those who responded to the follow-up survey, 55 (63%) indicated that they had not used the NMCD. Among this group, the most frequent reason cited for not using the tool was lack of awareness of the tool (n = 26; 50%). Seven additional respondents (n = 14%) indicated that while they had not personally used the database, they had asked a student or nurse to use the NMCD for them. Other reasons for not using the database were that no patients had inquired about H/NP (17 respondents), and two clinicians responded that they discourage all H/NP usage. Even among this group of individuals who had *not *used the H/NP database, 46 providers (85%) were of the opinion that the NMCD is a valuable tool when questions or concerns about dietary supplements arise. No demographic differences were apparent between respondents who had used the tool and those who did not. Of survey respondents who had used the H/NP tool, the greatest percentage of users was from the department of FCM (44% of survey respondents from FCM had used the tool).

Ninety-seven percent (31 of 32) of clinicians who had used the database found the tool improved their ability to find evidence-based information about H/NP, 91% reported that having the tool available increased their confidence when talking about H/NP with patients or colleagues, and 94% thought that the database had increased their overall knowledge of H/NP. Compared to respondents from the baseline survey, the electronic database significantly increased clinicians' ability to find reliable H/NP information (P < 0.0001), improved self-rated knowledge of H/NP (p < 0.0001) and increased clinician confidence when discussing H/NP with others (P < 0.0001) (Table 3).

**Table 3:** Perceptions of a Natural Product Database Tool: Before and After

	Pre N (%)	Post N (%)	P value
Would/did use the NMCD	54 (98)	32 (36)	< 0.0001
Would/did use for NMCD for patient safety (i.e. adverse effects)	44 (51)	20 (65)	P = 0.12
Would/did use NMCD to evaluate H/NP efficacy	45 (52)	27 (84)	P = 0.0033
Would/did use NMCD to identify ingredients in a trade name product	56 (64)	24 (75)	P = 0.0004
Would/did use NMCD to identify interactions	67 (77)	24 (77)	P = 0.1588
Would/did use NMCD to indentify contraindications	64 (74)	29 (90)	P = 0.1095
Uneasy discussing H/NP with patients/the NMCD has increased my confidence when talking about H/NP	35 (41)	29 (91)	P < 0.0001
Perceived knowledge of H/NP/the NMCD increased my knowledge of H/NP	32 (37)	30 (94)	P < 0.0001
It is easy to find reliable H/NP info/the NMCD improves ability to find reliable H/NP information	24 (28)	31 (97)	P < 0.0001

*A priori *perceptions of the utility of a H/NP tool and reported utilization. Data shown reflects affirmative responses; neutral responses were not included in analysis. Not all respondents answered every question.

As a result of having the NMCD available to look up H/NP, 63% of those who used the database indicated that they are now more likely to ask patients about H/NP when taking a drug history and the same percentage indicated that since having the NMCD tool, they have discussed H/NP with patients more often. Eighty-eight percent (28 of 32) of database users feel that having an evidence-based H/NP tool available has improved the quality of care that they provide to their patients.

Having the NMCD available to assist in evidence-based evaluations of H/NP also led to changes in clinical practice. Eleven respondents (33%) indicated that the database increased their likelihood of recommending H/NP to patients for medicinal purposes, whereas two respondents (6%) indicated that as a result of reading additional information about H/NP, the database decreased the likelihood that they would recommend an H/NP. Two respondents admitted that as a result of using the database, they personally started using H/NP, while one respondent indicated that s/he had personally stopped using a dietary supplement as a result of the tool.

Regardless of whether respondents had personally used the NMCD tool, 95% (83 of 87) indicated that the resource has potential to improve patient care. Overall, 12,337 page views on the NMCD originated from the PSU Hershey campus over the course of the year-long trial subscription, averaging more than 1,000 page views per month, and 81 of the 87 respondents (94%) were of the opinion that the initial trial subscription of the resource should continue. According to survey respondents, the most useful features of the NMCD pertained to efficacy data and identifying contraindications. Providers also relied upon the tool to identify individual ingredients in trade name products, identify adverse effects, and identify drug interactions (Table 3). Notably, more than 50% of clinicians had selected these items among their H/NP needs in the initial survey. Other frequently reported uses of the database included identifying appropriate doses of H/NP (19 of 33; 59%) and identifying putative mechanisms of action (22 of 33; 69%) for supplements. Nearly half of those who had used the database (47%) used the tool to print educational handouts for patients; additionally, three providers (9%) used the tool to identify United States Pharmacopeia (USP)-verified supplements to assure that high-quality products were recommended to patients.

## Discussion

Our study is the first to assess faculty attitudes, needs, and practice experiences with H/NP before and after implementing a solution to the H/NP needs that clinicians face. Furthermore, we are only the second group to explore physician acceptance, experiences, and knowledge of dietary supplements within an academic medical center [8,13,18].

Based upon responses obtained by our surveys, at our medical center, the NMCD tool appeared to be of greatest relevance to FCM providers. The openness and interest of FCM clinicians to nontraditional therapeutic alternatives may reflect a philosophical difference between FCM and other specialties, as this group of physicians tends to place great emphasis on developing, nurturing, and maintaining patient-centered relationships. Therefore, rather than simply disavowing use of all dietary supplements, it is important for FCM physicians to know about these alternatives in order to foster patient relationships. In contrast, our data found that our Med colleagues were less inclined to participate in our surveys and less likely to perceive H/NP knowledge as integral to the healthcare that they provide. This may reflect the role of internists as "specialists" as opposed to generalists. In the "specialist" culture, there is generally less emphasis on comprehensive care to patients across the lifespan, as the care is often limited by age, gender, or affected organ.

Our intent in this endeavor was not to create physician specialists in H/NP prescribing, but rather, was to simply examine changes in clinician attitudes and experiences toward dietary supplements when provided with access to a tool that could be utilized and applied at the point-of-patient-care. Based upon before and after comparisons of providers practicing in three clinical departments, we observed that access to the NMCD increased confidence when discussing H/NP with patients, increased self-reported knowledge of H/NP, and increased the likelihood of providers asking about H/NP when taking a drug history. We chose the NMCD because it had previously been found to be superior to other available electronic tools in terms of scope and completeness; additionally, it is reportedly the H/NP tool of choice by drug information centers [19-21].

Despite numerous faculty education sessions, demonstrations, and training modules that were created and delivered around the campus to demonstrate the utility of the NMCD, we were surprised that 63% of providers indicated on the follow-up survey that they were *unaware *that this resource existed. A subsequent questionnaire sent to all physicians inquired about the preferred method of communication about new resources; overwhelmingly, respondents indicated email as the preferred medium. We had extensively used e-mail to describe and to "advertise" the database and the opportunities that were available to learn more about this tool. Considering the deluge of email that is common, perhaps we should not be surprised at the poor communication afforded by this communication method. Communication difficulties regarding new endeavours are apparently not unique at our institution [22]. Wahner-Roedler et al. [23] reported that 49% of physicians at Mayo Clinic found it difficult to find reliable information about herbs, despite the institution having subscribed to a web-based resource for many years. Collectively, these experiences demonstrate the need for better intra-institutional methods of communication.

Limitations to our evaluation exist. First, the response rate among providers at our institution was lower than anticipated. Our response rate was similar, however, to that of Chan and Wong, in their evaluation of physician attitudes, practices, and training with respect to a wide range of complementary and alternative medicine (CAM) practices [24]. These low response rates may reflect an unspoken perception that CAM, including H/NP, does not have a place within academic medical centers. Given that we do not have access to demographic data from the non-responders, we do not know if any distinguishing characteristics (gender, age, training, etc.) would differentiate responders from non-responders. Nonetheless, our response rate was nearly identical for both the baseline assessment and the follow-up. Given our response rate, self-selection bias may have occurred, leading to over-representation of responses among those with the strongest feelings (pro or con) about H/NP. Another limitation to the dataset may have occurred with respect to the knowledge assessment. It is conceivable that inclusion of the "I don't know" option may have decreased overall knowledge scores. While our intent by including this selection was to limit provider "guessing", it is possible that responders may have selected this response in haste to complete the survey instrument. Nonetheless, we believe it is probable that our clinicians truly did not know the correct answers to the knowledge questions about commonly-used H/NP since similar knowledge scores have been identified at other institutions [23].

We recognize that the initiative that we described may not be necessary at institutions that have Integrated Health Centers (and presumably a wider array of CAM resources). Nonetheless, for medical centers such as ours, the NMCD can be a valuable tool. In addition, we recognize that implementing clinical decision tools such as the NMCD can be limited by cost at some medical centers. Economic and financial aspects are important considerations, yet, we believe that the utility of such a tool can outweighs the economic impact.

## Conclusions

In conclusion, we have found that using a web-based resource to find accurate information about H/NP in the context of patient care has improved clinician confidence discussing H/NP, increased the likelihood that providers inquire about H/NP when taking drug histories, and increased clinicians' perception of the quality of care they can provide to patients. With the dynamic environment that surrounds consumer use of H/NP, electronic decision tools may be a critical element to meet clinical needs. Patients that visit academic medical centers tend to have complex medical problems and chronic illnesses; they are precisely the population of patients that resort to using H/NP when they perceive that conventional medicine has failed them. In academic settings, it may be especially important for clinicians to consider the impact of H/NP in patient care. Our data indicate that providers do not necessarily need to master additional coursework in the area of H/NP; simply having access to an evidence-based H/NP decision tool can aid therapeutic discussions and inform decisions that can rationally guide patient care.

## Competing interests

The authors declare that they have no competing interests.

## Authors' contributions

KK conceived of the study, designed the study and drafted the manuscript. SB conducted statistical analysis of the data and revised the manuscript. Both authors have read and approved the final manuscript.

## Authors' information

KK, RPh, PhD, serves as director of pharmacology medical instruction at Penn State-Hershey where she educates students and healthcare providers to maximize health outcomes and minimize adverse events through responsible medication utilization.

## Pre-publication history

The pre-publication history for this paper can be accessed here:

http://www.biomedcentral.com/1472-6963/11/279/prepub
